# Short-term forecasts and long-term regional scenarios of climate change effects on Swedish forest phenology

**DOI:** 10.1007/s00484-026-03259-5

**Published:** 2026-07-06

**Authors:** Ola Langvall, Gustav Strandberg

**Affiliations:** 1 Swedish University of Agricultural Sciences, Siljansfors Experimental Forest, Mora, Sweden; 2 Swedish Meteorological and Hydrological Institute, Norrköping, Sweden; 3 Department of Thematic Studies – Environmental change, Centre for Climate Science and Policy Research, Linköping University, Linköping, Sweden

**Keywords:** Phenology, Forecast models, Climate change scenarios, Bark beetle swarming, Budburst, Ripe berries

## Abstract

Comprehensive forest phenology data from the Swedish National Phenology Network has been used to produce forecast models of the seasonal phenology on tree species, wild berries and insects, which are made available to the public. One model forecasts the onset, maximum appearance and ending of the budburst and leaf/shoot elongation of the most common tree species in Sweden; Silver birch (*Betula pendula* Roth), Downy birch (*B. pubescens* Ehrh.), Norway spruce (*Picea abies* [L.] H. Karst.) and Scots pine (*Pinus sylvestris* L.). Furthermore, a model for the onset, maximum appearance and ending of flowering and ripening of the most common wild berries in Sweden; Cowberry (*Vaccinium vitis-idaea* L.) and Bilberry (*V. myrtillus* L.), and a model for the timing of the swarming of the European spruce bark beetle (*Ips typographus* Linnaeus, 1758) for the mother generation and, more interestingly, for the coming generations during the same season, are also available. Daily air temperature data from the sites where phenology observations has been undertaken, is used to produce critical accumulated temperatures for when target phenological phases appear. Current year’s weather data, for estimation of the current status, and long-term averages, for predicting the near future, are used to produce seasonal forecasts predicting the timing of e.g. budburst, berry ripening and swarming of first-generation offspring that may establish a second generation of beetles. The assigned critical accumulated air temperatures for phenological phases to appear in the forecast models have also been applied on scenario temperature data, to explore possible long-term effects of climate change in the Swedish forests. The scenarios predict broadleaf trees to have a 9–41 days earlier start of the season in the period 2070–2099, compared to the reference period 1970–1999, depending on the applied scenarios (average of all sites and species, shortest for RCP2.6 and longest for RCP8.5). A general pattern is that the spruce bark beetle may be able to produce two new generations on one season at the end of the century, in the south this can be the new normal, in the north only occasionally and only under the strongest climate change scenario (RCP8.5). Northern Sweden and local continental areas are expected to experience the least change, compared to southern Sweden and, especially, the maritime west coast area, at least for some of the tested traits.

## Introduction

Phenology has become one of the ways to display how climate change affects nature. It is also a way to reach out to the public, as many already are engaged in checking for the signs in nature that show phenological changes during the seasonal changes we experience throughout the year, like the first spring flower, butterfly or bumble bee, or autumn colours of broadleaved trees that are found in some areas around the world.

Phenological signs have always been part of the observations made by humans, to be able to accommodate their lives to the coming seasons. It has been popular to set up calendars that predict the timing of certain activities, e.g. in agriculture – where you like to plan when it is time to plow the fields and to harvest. Also in modern times, the need to know when certain phenological traits occur in nature is evident. It is now evident that the weather varies from year to year, so a fixed calendar will not work as a valid tool to plan activities in nature in detail, especially considering the high variation there is in the local climate of a country like Sweden, with a latitudinal range from 55°N to 69°N, altitudinal range of -2.3–2096.8 m a.s.l, and a strong gradient from local maritime to continental climate.

The predicting models described in this paper have been developed on request from other research studies or from forest-related companies and agencies that benefit from “knowing” in advance when the phenological traits appear, to be able to plan their activities related to it. A few examples are (a) when aerial photos and aerial laser scanning should be taken, depending on whether you like to include leaf-out on broadleaf trees or not, (b) the planning of harvest of wind-thrown Norway spruce, to avoid massive invasion of European spruce bark beetle, (c) the timing of Bilberry (*Vaccinium myrtillus* L.) flowering, when studying the appearance and behaviour of pollinating insects, or (d) the timing of ripe forest berries in different parts of the country, to be able to plan seasonal staffing and the travel routes for them, in professional berry picking companies.

Furthermore, for the long-time planning, there is also a need to be able to predict how Nature’s calendar will look like, both globally but also on a local scale, in the future climate. Various attempts to visualise the future climate has been made and currently, the most commonly used scenarios when modelling it are the RCP scenarios (Moss et al. 2010; van Vuuren et al. 2011). The RCP2.6 scenario assumes that greenhouse gases decrease fast to reach net-negative values in the end of the century. The RCP4.5 scenario assumes that greenhouse gas emissions will culminate around 2040, to then fall to half the values by 2100. Finally, the RCP8.5 scenario assumes increasing emissions until year 2100. The global temperature increases between 1986 and 2005 and 2081–2100 is on average 1.0 °C (RCP2.6), 1.8 °C (RCP4.5) and 3.7 °C (RCP8.5), respectively (IPCC 2013). Each of the scenarios has been used in climate model simulations within the CORDEX programme (Coordinated Regional Climate Downscaling Experiment; Jacob et al. 2014). Several different global climate models (GCM) have been used to drive several different regional climate models (RCM). Combining the models in so called model ensembles offers ways to both see the general trend and the effect of the scenario assumption, and to assess the robustness of the result by comparing the spread between ensemble members.

The aims of this study were to (i) document the principal components of the forest phenology forecast models, and (ii) to evaluate the change in timing of these phenology traits, due to a changed climate.

## Materials and methods

### Locations

**Fig. 1:**
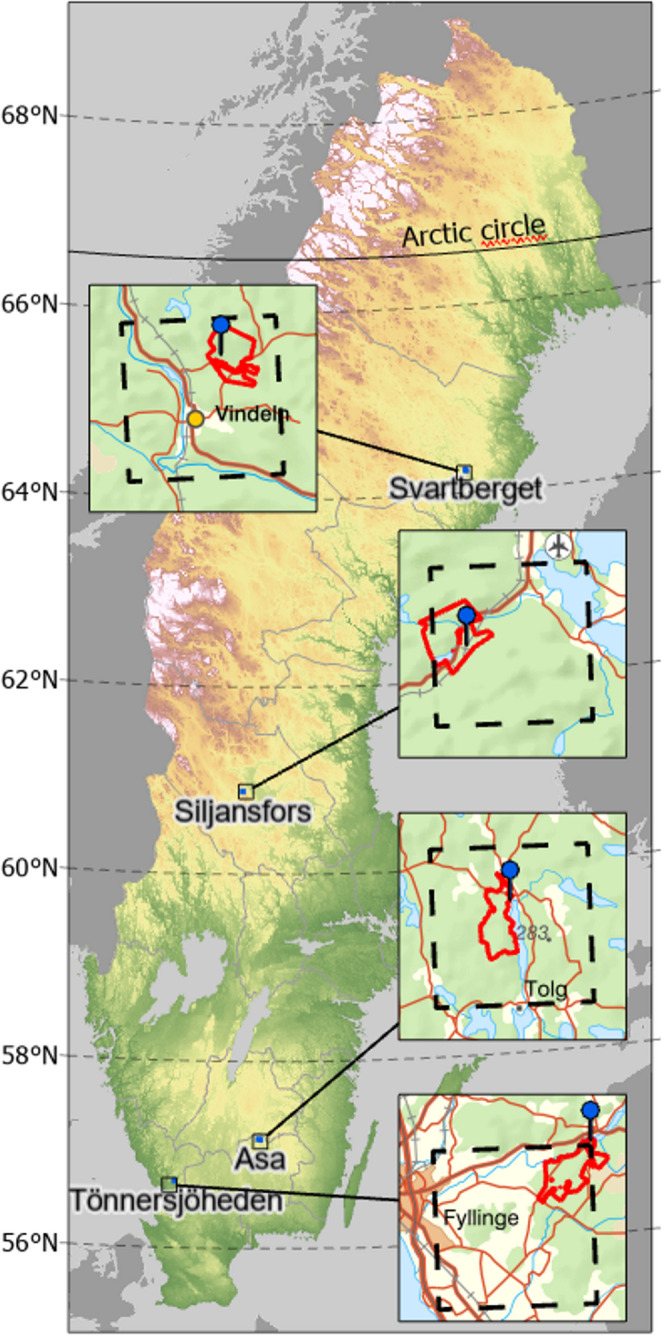
Locations of sites for phenological assessments (i.e. experimental forests, red polygons), reference climate stations (blue pins) and local scenario grid cells (black hash squares)

This study was made for four sites in Sweden, with a latitudinal range 56.7–64.2°N and a climatological range from local maritime to local continental (Table 1; Fig. 1). The continentality may be described as a combination of altitude and distance to the sea or other big water bodies (McIlveen 1992).

**Table 1:** Experimental forests in Sweden, where the phenology assessments and local climate measurements were performed, and to where the gridcell of scenario data was extracted

Experimental forest	Start year	Position				Temperature climate 1991–2020		
		Latitude	Longitude	Altitude	Distance to sea	Annual avg.	Accumulated (thresh. +5 °C)	Growing season
				m	km	°C	Degree-days	Days
Svartberget	1923	64°14´N	19°46´E	160–320	59	2.4	977	150
Siljansfors	1921	60°53´N	14°24´E	210–425	150	4.0	1 121	167
Asa	1988	57°10´N	14°45´E	165–286	78	6.5	1 417	201
Tönnersjöheden	1923	56°42´N	13°06´E	60–140	17	7.6	1 553	211

### Phenology

Weekly ground observations of phenology included in this study has been made at the four sites during 2006–2020 (Langvall 2021; Langvall and Ottosson Löfvenius 2019).

In a forest stand suited for each of the berry species Cowberry (*Vaccinium vitis-idaea* L.) and Bilberry (*V. myrtillus* L.), 9–10 circular plots of 0.28 m radius (i.e. 0.25 m²) were established on each site. The size of the plots was selected to match the plots applied in the Swedish National Forest Inventory, as these assessments also were used for estimating total berry production in the Swedish forests. The size and numbers of plots were chosen to receive a reasonable workload and accuracy of the estimated numbers and the variation in numbers in the forest. The plots were arranged to be at least 10 m apart in the stand, to avoid assessing the same clone. On each plot, the number of open flowers, unripe and ripe berries of the target species were counted. Assessments were made weekly from before the first flowering until most of the berries are gone or winter conditions are emerging.

**Fig. 2:**
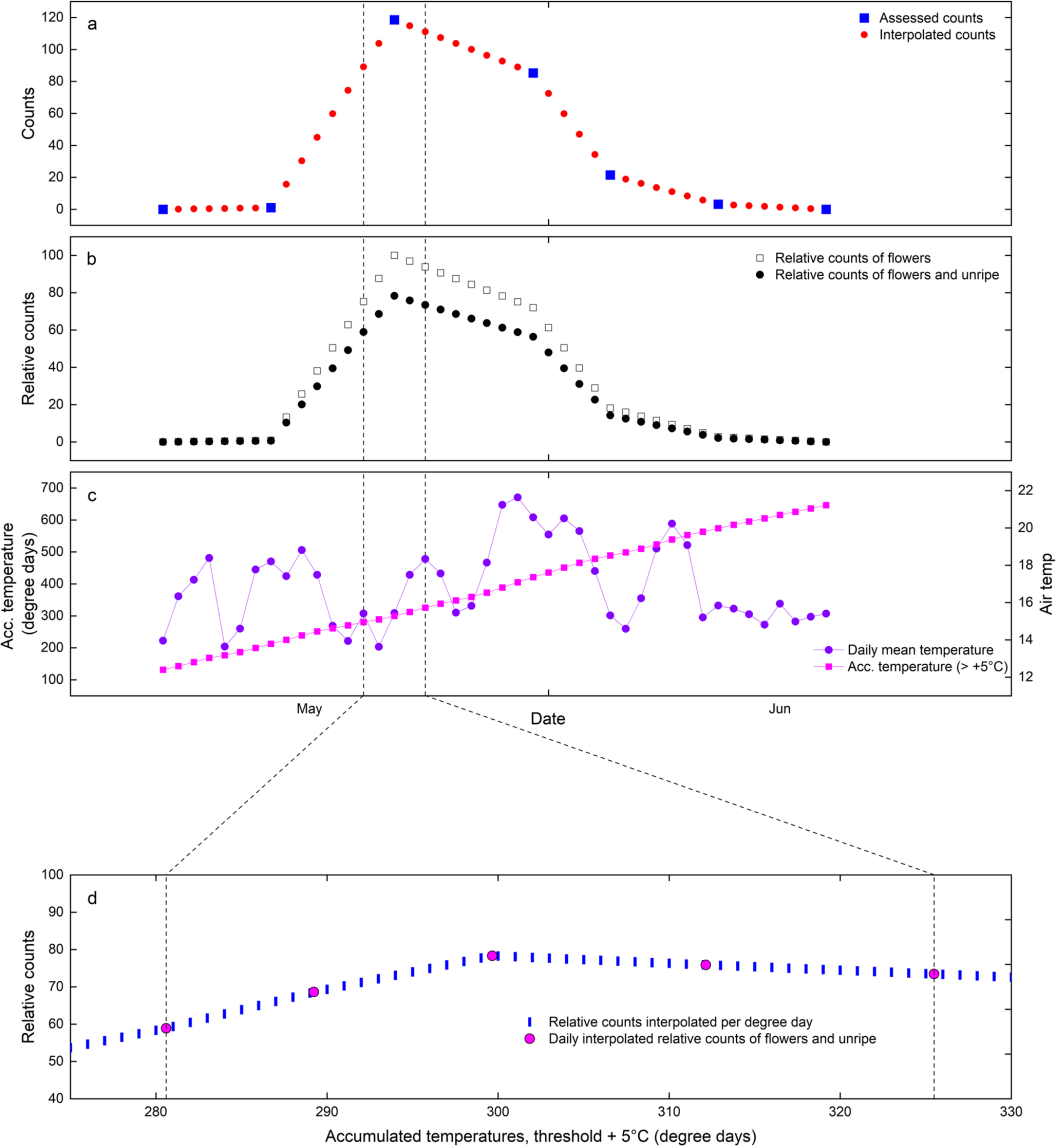
Example of calculations from data assessed at the Tönnersjöheden site (57°N) in 2018, (**a**) average assessed counts per m^− 2^ of bilberry flowers and interpolated counts between assessment days, (**b**) interpolated counts relative to maximum counts of flowers and flowers + unripe berries (= total numbers), respectively, per day, (**c**) daily mean air temperature and accumulated temperatures (threshold + 5 °C) from Jan 1^st^ 2018, and (**d**) daily relative counts of flowers vs. accumulated temperature and interpolated relative counts of flowers for each degree day of accumulated temperatures

The assessments of single plots were averaged to the mean number of open flowers, unripe and ripe berries per square meter at each assessment day, for each berry species and site (Fig. 2a). In turn, these were linearly interpolated between assessment days, to achieve daily estimates of the counts of flowers and ripe berries during the growing season (Fig. 2a). Finally, relative counts were assigned, where the interpolated daily counts were compared to the maximum number of flowers and ripe berries for the season, to form a value 0–100% for each day (Fig. 2b). The relative number of berry flowers was estimated as the fraction of flowers of the maximum total of flowers + unripe berries (= the estimated total numbers of flowers during the season), while the relative number of ripe berries was estimated as the fraction of ripe berries of the total of the maximum of unripe + ripe berries (= the estimated total numbers of ripe berries during the season).

**Fig. 3:**
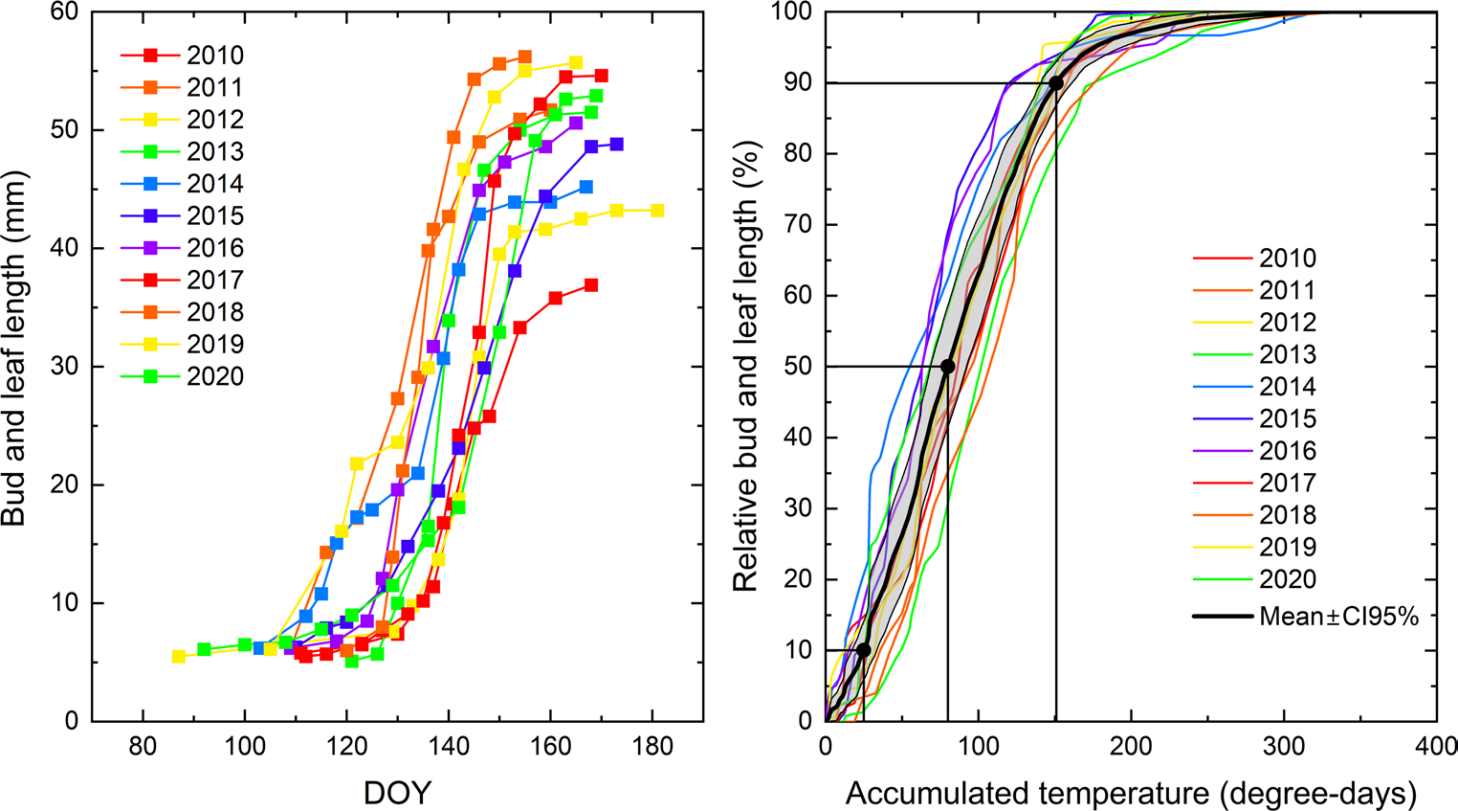
Phenological assessments on Silver birch in Siljansfors (61°) during 2010–2020: Absolute length of buds and leaves on assessment days (left) and normalised bud and leaf length (relative to final length the same year) vs. achieved accumulated temperature (threshold temperature + 5 °C) on the assessment days (right). Mean critical accumulated temperatures for 10, 50 and 90% leaf length are used in the phenology forecast models (black dots)

Furthermore, one bud was marked on five Silver birch (*Betula pendula* Roth) and five Downy birch (*B. pubescens* Ehrh.) trees per site, before the start of the season. The length of the bud and, after flushing, the emerged leaf was measured with a calliper. Assessments were made weekly from before the start until the leaf reached full length, *i.e.* when the same length was measured two weeks in a row (Fig. 3).

Similarly, the apical bud was marked on five Scots pine (*Pinus sylvestris* L.) trees per site. Bud length, and after budburst, the shoot length was measured with a calliper. Assessments were made weekly from before the start until the shoot reached full length.

The apical bud was also marked on five Norway spruce (*Picea abies* [L.] H. Karst.) trees per site. Bud and shoot development was assessed using the phenological classification defined by Krutzsch (1973). Assessments were made weekly from before the start until the shoot reached full length, i.e. when the shoot reached stage 8 (full length reached and new buds are formed along the axis).

The assessments of single trees were averaged to a mean phenology development stage at each assessment day, for each tree species and site. In turn, these were interpolated between assessment days, to achieve daily estimates of their phenology development during the growing season. Finally, a relative development stage was assigned, where the daily estimate of bud/leaf/shoot size (birches and Scots pine) was compared to the initial and final size, and for Norway spruce relative to the Krutzsch classification (0–8), to form a value 0–100% for each day.

Finally, the European spruce bark beetle (*Ips typographus* Linnaeus, 1758) was collected at 5 locations within an area of 20 km² near the sites. Collection was made with three Nove traps per location, provided with a pheromone to actively attract the beetles. The traps were assessed weekly May–September, i.e. during the period when swarming of the mother generation (generation 0) and first generation (generation 1) can be caught in the traps.

Critical accumulated temperatures for the peak of the initial swarming of the mother generation, and for the period between this swarming and the next generation’s swarmings were derived from an extensive dataset of collected bark beetles from several sites throughout Sweden, performed within the national monitoring program, run from the Swedish Forest Damage Center, at the Swedish University of Agricultural Sciences in Uppsala (Fritscher and Schroeder 2022).

### Climate data

Climate stations have been established on or near the four sites early in the history of the experimental forests (Table 1), in most cases as part of the national network of weather stations, run by the Swedish Meteorological and Hydrological Institute (SMHI). The earliest establishment was made at the Siljansfors Experimental Forest, in 1921.

**Fig. 4:**
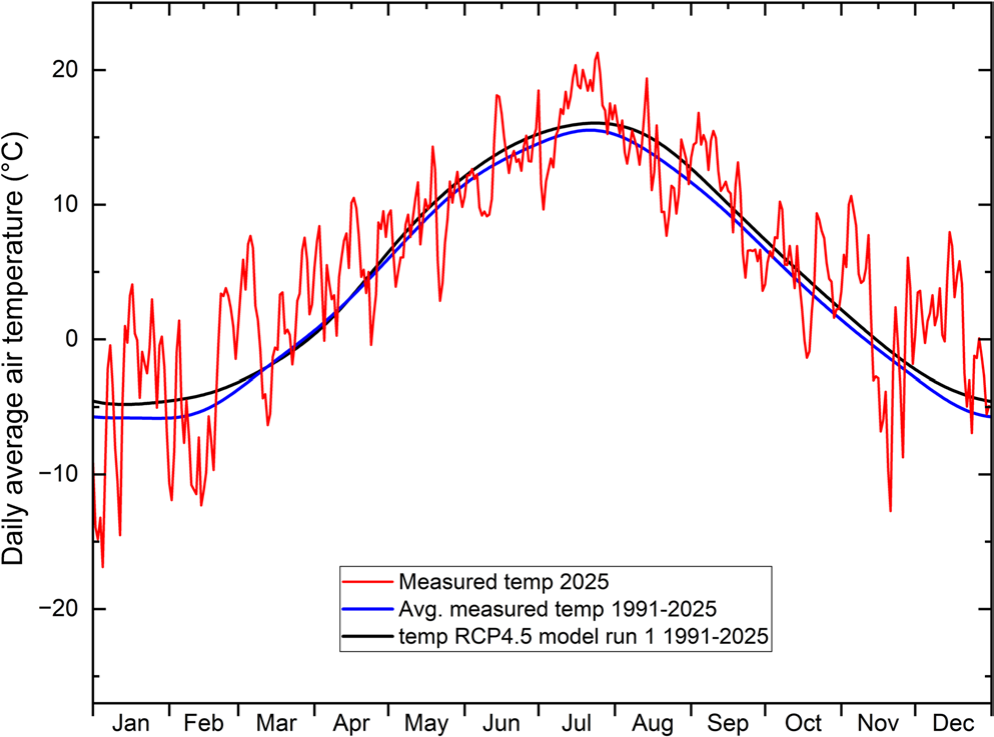
Daily average air temperature at screen height at the site Siljansfors (61°), measured on site during 2025 (red), average site measurements for the period 1991–2025 (blue), and simulated average temperature for the period 1991–2025 by the scenario RCP4.5 model run 1 (black)

A common program for reference climate monitoring was established at all four sites in 1990. From then, air temperature is monitored with shielded and ventilated temperature sensors at 1.7 m height. Temperature readings were collected every 1 min with Campbell dataloggers and aggregated to 10-minute and daily average outputs (Fig. 4). Data has been quality checked, before made available online (SLU 2026a). Today, data collection and storage are made to a database continuously, i.e. raw data is available for assessments of the forecast models in near-present time.

Calculations of future climate are made using a sub-set of CORDEX simulations. To ensure a consistent comparison of scenarios, only models available for all emission scenarios (RCP2.6, RCP4.5, RCP8.5) were selected. This resulted in 17 simulations for each scenario. Using all available simulations would result in model ensembles of different sizes for different scenarios. In that case it is difficult to know if the difference between scenarios is because of different emissions or because different models, and different number of models, are used. The simulations are made on a horizontal resolution of 12.5 × 12.5 km and for the period 1970–2099. To minimise systematic errors, bias adjustment was applied, using the MIdAS method (Berg et al. 2022). This model ensemble is described in more detail in Strandberg et al. (2026).

### Calculations

All calculations and statistical analyses were made using R (R Core Team 2025), including the dplyr package (Wickham et al. 2023).

The quality checked daily average temperatures from each site were accumulated daily, starting at zero on January 1^st^ every year (Fig. 2c). As daily average temperatures generally are sub-zero or near zero at these latitudes in January, we find it appropriate to start accumulating temperatures on this date, while a later starting date could lead to missing early warm spells. The threshold temperature was set to + 5 °C for the simulations of trees and berry shrubs. This threshold temperature was used as it is a commonly used threshold when defining the growing season as a general climate indicator (Strandberg et al. 2024). As we did not have specific studies of these trees and scrubs showing an individually optimized selection, we decided this was our best guess. Also, in this study, the accumulated temperature can be seen as a proxy of the temperature climate defining when a phenological trait occurs, rather than an absolute number.

Accumulated temperature (*T*_*a, doy*_) for a specific day of the year (*doy*) and threshold temperature (*t*_*threshold*_) was calculated as

$$\:{T}_{a,doy}= \sum_{i=1}^{doy}({t}_{i}-{t}_{threshold})\cdot\:I({t}_{i}>{t}_{threshold})$$

where *I(t*_*i*_ *> t*_*threshold*_) is an indicator function that equals to 1 when the condition is satisfied and 0 otherwise.

The datasets with daily relative phenology estimates were associated with the datasets of daily accumulated temperatures from each site. To achieve a relative phenology estimate for each degree of accumulated temperatures, the relative phenology stages where linearly interpolated between the accumulated temperatures with known phenology stages (Figs. 2d and 3). The average of all available year’s estimated relative phenology stage for each degree of accumulated temperatures was calculated and, finally, the average critical accumulated temperature for each target phenology phase and stages were extracted, together with the 95% confidence interval of the relative phenology stages at the average critical accumulated temperatures (Table 2).

**Table 2:** Critical accumulated air temperatures, specified for the species, phenology phase and stage used in this study, and the 95% confidence interval of the relative pheno-phase stage at the specified critical accumulated air temperature

Species	Pheno-phase	Stage	Threshold temperature	Critical accumulated air temperatures, degree-days (95% CI of relative pheno-phase stage, %)							
			°C	Svartberget		Siljansfors		Asa		Tönnersjöheden	
Downy birch	Budburst	10%	5.0	33	(± 2.7)	52	(± 3)	64	(± 4.3)		
		50%	5.0	98	(± 5.2)	118	(± 5.4)	138	(± 7.7)		
		90%	5.0	182	(± 2.1)	194	(± 2.6)	239	(± 6.2)		
Silver birch	Budburst	10%	5.0			25	(± 4.1)	41	(± 3.8)	49	(± 4.1)
		50%	5.0			80	(± 8.5)	109	(± 9.1)	105	(± 12.6)
		90%	5.0			152	(± 3)	204	(± 5.8)	168	(± 9)
Norway spruce	Budburst	10%	5.0	69	(± 1.8)	116	(± 3.9)	88	(± 4.3)	146	(± 3.4)
		50%	5.0	189	(± 3.8)	229	(± 6.8)	257	(± 7.3)	293	(± 4.9)
		90%	5.0	340	(± 4)	417	(± 3.3)	460	(± 7.4)	572	(± 4.4)
Scots pine	Budburst	10%	5.0	80	(± 1.8)	94	(± 2)	114	(± 1.6)	106	(± 1.3)
		50%	5.0	185	(± 5.2)	208	(± 4.5)	242	(± 4.9)	220	(± 3.9)
		90%	5.0	277	(± 2.7)	328	(± 3)	422	(± 5.9)	366	(± 3.1)
Bilberry	Flowering	> 10%	5.0	74	(± 7.6)	103	(± 11.3)	81	(± 8.4)	81	(± 7.7)
		Max	5.0	141	(± 10.4)	116	(± 12)	132	(± 17.4)	142	(± 14)
		< 10%	5.0	251	(± 9.1)	214	(± 5.2)	264	(± 4.7)	305	(± 14.1)
	Ripe	> 10%	5.0	558	(± 4.9)	534	(± 3)	554	(± 5.1)	541	(± 3.5)
		Max	5.0	713	(± 7.1)	769	(± 5.9)	758	(± 13)	696	(± 14.1)
		< 10%	5.0	955	(± 5.6)	1140	(± 5.4)	1272	(± 5.1)	1106	(± 9.9)
Cowberry	Flowering	> 10%	5.0	247	(± 4.2)	190	(± 9.7)	163	(± 15.6)	198	(± 10.2)
		Max	5.0	328	(± 12.3)	292	(± 9.4)	342	(± 14.1)	328	(± 13.3)
		< 10%	5.0	461	(± 9.5)	439	(± 6.2)	568	(± 24.4)	527	(± 8.4)
	Ripe	> 10%	5.0	793	(± 6.2)	818	(± 5.2)	1031	(± 6.9)	933	(± 6.8)
		Max	5.0	989	(± 11.4)	1025	(± 9.1)	1179	(± 10.5)	1156	(± 9.7)
		< 10%	5.0	1129	(± 7.1)	1213	(± 7.3)	1397	(± 6.7)	1381	(± 6.3)
Bark beetle	Generation 0		8.3	39		30		37		37	
	Generation 1		8.3	459		450		537		537	
	Generation 2		8.3	879		870		1037		1037	
	Generation 3		8.3	1299		1290		1537		1537	

For tree phenology, the target phenology stages were bud/leaf/shoots reaching 10, 50 and 90% relative size, as indicators of the start, halfway and end of flushing, respectively. For berry phenology, the target phenology stages were when the relative number of flowers and ripe berries reached 10%, its maximum, and when only 10% remains, respectively, indicating the start of the flowering/picking period, when maximum flowers/berries are available, and when the end of flowering/picking period is close.

### Forecast models

The input data for the forecast models is formed from two datasets of the local climate data for each site. One dataset (DS_avg_) consists of a long-term (+ 30yrs) mean of quality checked daily average temperatures from 1990 until the year before current year. The other dataset (DS_curr_) consists of the current year’s daily average temperatures until the day before current day.

**Fig. 5:**
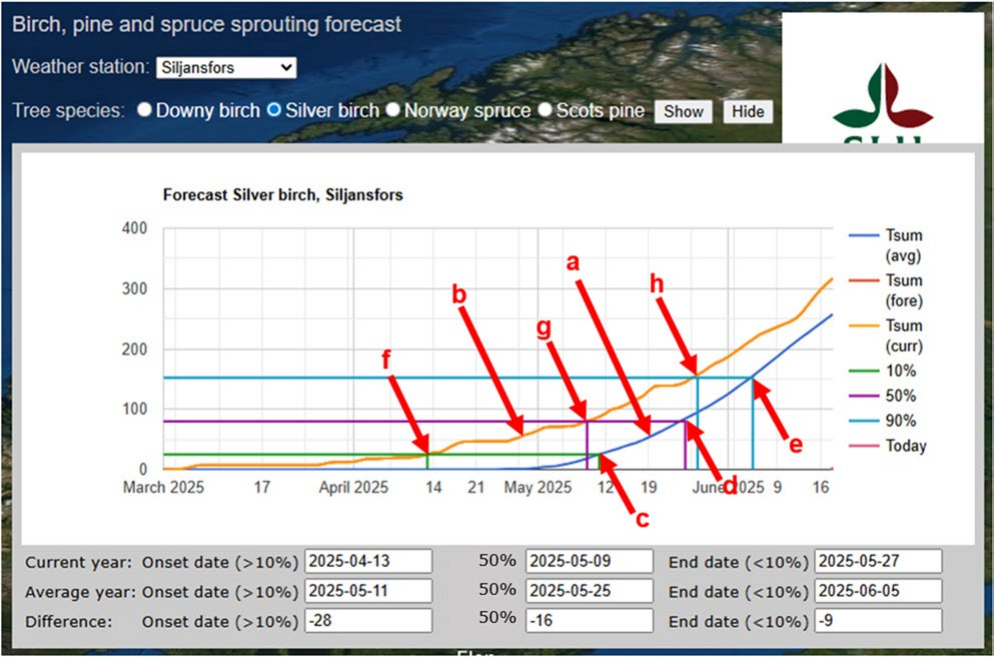
Illustration of the phenology forecast model for forest trees, as performed for Silver birch in Siljansfors (61°N) for the year 2025. The graph shows the accumulated temperatures (threshold temperature + 5 °C) based on the mean of measured daily average temperatures during the years 1991–2004 (**a**) and on the current year daily average temperatures (**b**). The dates for reaching the critical accumulated temperatures for 10, 50 and 90% leaf length if temperatures never diverge from the average of the preceding 30 + years (**c**, **d** and **e**) and the current year (**f**, **g **and **h**) are also shown in the graph and in the table below, together with the number of days’ difference

For forecasts of the timing of leaf and shoot development of Scots pine, Norway spruce, Downy birch and Silver birch, and flowering and ripening of forest berries, DS_curr_ was used to calculate the already achieved accumulated temperature (threshold temperature + 5 °C) for each day until the day before present day, the current year, and DS_avg_ was used to forecast the accumulated temperatures each day for the rest of the season, simulating that the future weather will not deviate from the average temperatures achieved in DS_avg_. The dates when the critical accumulated temperatures for each phenology stage are displayed in the graph, as well as the defined date, on the web site (Fig. 5; SLU 2026b, c). Also, the daily accumulated temperatures and dates when critical stages were passed, if the whole season would have developed as if temperatures never deviated from the average temperatures in DS_avg_, together with the deviation between current-year and “normal” year conditions, is displayed for comparison.

For forecasts of the timing of the swarming of Spruce bark-beetle generations, DS_curr_ was used to calculate the already achieved accumulated temperature (threshold temperature + 8.3 °C) for each day until the day before present day, the current year, and DS_avg_ was used to forecast the accumulated temperatures each day for the rest of the season, simulating that the future weather will not deviate from the average temperatures achieved in DS_avg_. The dates when the critical accumulated temperatures for swarming of each bark beetle generation are displayed in the graph, as well as the defined date, on the web site (SLU 2026d). Also, the daily accumulated temperatures and dates when critical accumulated temperatures for swarming were passed, if the season would have developed as a “normal” year, is displayed, together with the deviation between current-year and “normal” year conditions, is displayed for comparison.

Note that the forecast models are not statistical models, but models where the extracted critical accumulated temperatures from phenology observations on the sites and species has been applied on the current year and an average year daily average temperatures for each site.

### Scenarios

For the scenario evaluations, 17 model runs of the regional assessment data of climate scenarios RCP2.6, RCP4.5 and RCP8.5 where used. Accumulated temperatures, starting on January 1^st^ every year, were calculated for each model run.

The critical accumulated temperatures assigned for each phenology phase and stage were applied to these accumulated temperatures, to achieve the day of the year (DOY) when the stage was reached, for each year and model run. An average yearly DOY for all model runs on the same phenology phase and stage was also calculated. To compare simulated historic time phenology with the future, considering the modelled climate change implemented in the three scenarios, the average DOY for the period 1970–1999 was compared with the average DOY for the period 2070–2099.

To compare the modelled scenarios for the future with historical phenology, the critical temperatures for some of the phenology phases and stages were also applied to yearly accumulated historical records of temperatures for the sites. The average DOY for the period 1970–1999, based on measured temperature data was also compared to the modelled average DOY of the same period, based on scenario data.

## Results

### Forecast models

Generally, the critical accumulated temperatures achieved from the phenological observations were lower towards higher latitudes. Exceptions were found for the berry pheno-phases and the swarming of spruce bark beetle, which were also affected by the continentality of the site, especially pronounced for the Siljansfors site (Table 2; Fig. 3).

We have been able to display the current year deviation from a “normal” year of the development of the accumulated temperatures during the full season at a site, by applying historically measured temperatures and the current year temperatures. When applying the critical temperatures on historically measured temperatures and the current year temperatures, we have been able to forecast the dates when the pheno-phase will presumably occur, both during the current year and for an average year. In the example in Fig. 5, the leaves of Silver birch were forecasted to reach 50% of its final size in May 9 during 2025, as compared to May 25 a normal year, i.e. the development of the leaves advanced 16 days this year, due to an unusually early spring. The final size was reached only 9 days ahead of the normal date, as the early spring was followed by a rather cold May (Fig. 4).

### Scenario modelling

**Fig. 6:**
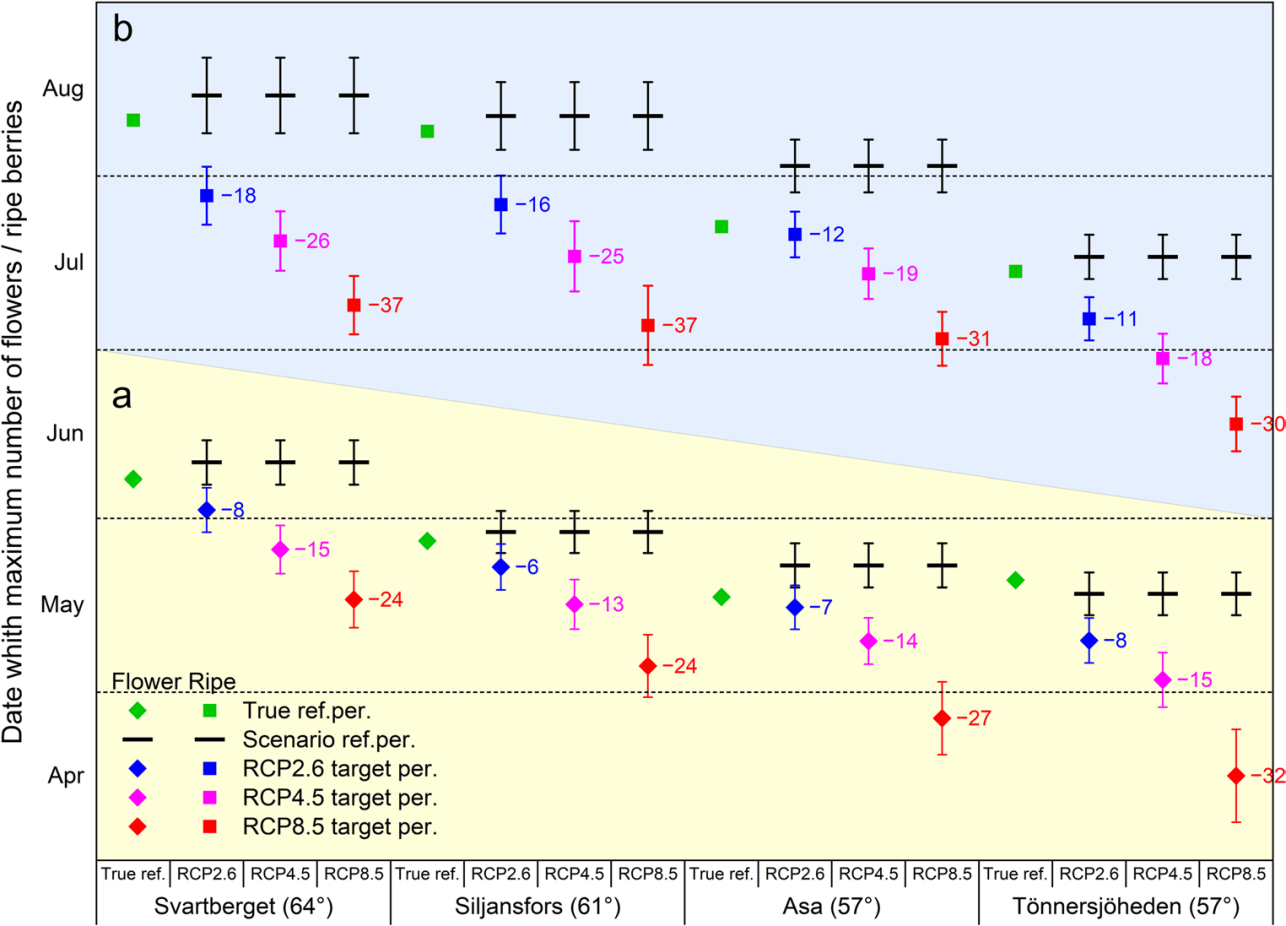
Average dates when maximum numbers of Bilberry flowers (**a** – light yellow background) and ripe berries (**b** – light blue background) occur, as assessed during 2006–2020 (green), and as estimated according to three locally adapted climate scenarios (RCP2.6, RCP4.5 and RCP8.5) for the reference period 1970–1999 (black) and the target period 2070–2099 (blue, violet and red), for the four Swedish sites. Bars show the 95% confidence interval for all model runs used for each scenario, and labels show the number of days’ deviance for the mean date of the target period from the reference period

**Fig. 7:**
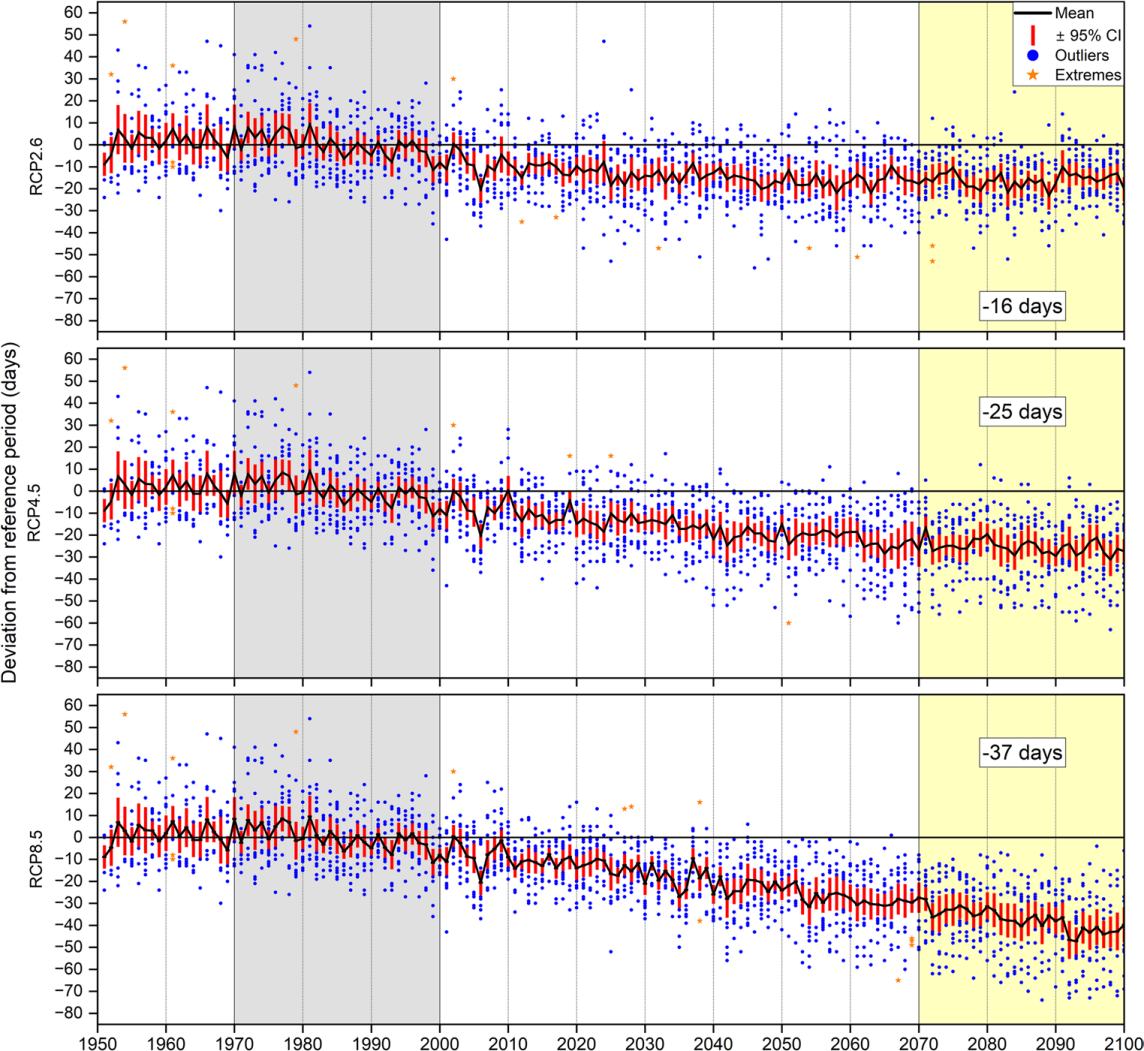
Deviation from the mean date for the reference period 1970–1999 when maximum numbers of Bilberries are estimated to occur at the site Siljansfors (61°), according to three locally adapted climate scenarios, RCP2.6 (top), RCP4.5 (middle) and RCP8.5 (bottom). Black lines show the moving average (5 years), red bars show the 95% confidence interval (CI) of all model runs each year (*n* = 17) and dots show single model run results exceeding 95% CI

When applying the critical accumulated temperatures on the temperature scenarios, the trend was that the date of all phenological traits were advanced by a few or more days (Table 3). As expected, the lowest numbers were found when assessed on the climate scenario RCP2.6, as this was modelled for the smallest temperature changes among the applied scenarios, and the highest numbers for the scenario RCP8.5 (Fig. 6). The variation between the different model runs of the same scenario was evident, but when averaging the dates of all 17 runs, the trend was clear and showed a rather low confidence interval for the yearly estimates. For example, the maximum of ripe Bilberries in Siljansfors was estimated to occur on average 16, 25 and 37 days earlier during the period 2070–2099, compared to the period 1970–1999, under the temperature conditions modelled for the scenarios RCP2.6, RCP4.5 and RCP8.5, respectively (Fig. 7).

**Table 3:** Mean dates for all species, phenology phases and sites to occur during the reference period (1970–1999) and target period (2070–2099), for 17 model runs of three locally adapted scenarios (RCP2.6, RCP4.5 and RCP8.5), and the difference from the target to the reference period

Species	Phenology phase	Site	Ref. per. (1970–1999)	Target period (2070–2099)					
				RCP26		RCP45		RCP85	
			Date	Date	(diff)	Date	(diff)	Date	(diff)
Silver birch	Budburst 10%	Siljansfors	May 8	Apr 30	(-8)	Apr 19	(-20)	Mar 28	(-41)
		Asa	May 3	Apr 23	(-11)	Apr 13	(-21)	Mar 14	(-51)
		Tonnersjoheden	Apr 29	Apr 15	(-15)	Apr 3	(-27)	Mar 1	(-60)
	Budburst 50%	Siljansfors	May 22	May 16	(-6)	May 10	(-13)	Apr 28	(-25)
		Asa	May 19	May 12	(-8)	May 5	(-14)	Apr 20	(-29)
		Tonnersjoheden	May 12	May 3	(-10)	Apr 25	(-18)	Apr 3	(-39)
	Budburst 90%	Siljansfors	Jun 4	May 28	(-7)	May 21	(-14)	May 10	(-25)
		Asa	Jun 3	May 26	(-8)	May 20	(-15)	May 8	(-26)
		Tonnersjoheden	May 22	May 14	(-9)	May 7	(-16)	Apr 22	(-31)
Downy birch	Budburst 10%	Svartberget	May 20	May 12	(-8)	May 6	(-15)	Apr 26	(-25)
		Siljansfors	May 16	May 10	(-7)	May 3	(-13)	Apr 19	(-28)
		Asa	May 10	May 2	(-8)	Apr 24	(-17)	Apr 1	(-39)
	Budburst 50%	Svartberget	Jun 3	May 26	(-9)	May 19	(-15)	May 11	(-24)
		Siljansfors	May 29	May 23	(-7)	May 16	(-13)	May 6	(-24)
		Asa	May 24	May 17	(-8)	May 11	(-14)	Apr 27	(-28)
	Budburst 90%	Svartberget	Jun 16	Jun 7	(-9)	May 31	(-17)	May 22	(-26)
		Siljansfors	Jun 10	Jun 2	(-8)	May 26	(-16)	May 16	(-26)
		Asa	Jun 8	May 31	(-8)	May 25	(-15)	May 13	(-26)
Scots pine	Budburst 10%	Svartberget	May 31	May 23	(-8)	May 16	(-15)	May 7	(-24)
		Siljansfors	May 25	May 19	(-7)	May 13	(-13)	May 1	(-24)
		Asa	May 20	May 13	(-8)	May 6	(-14)	Apr 22	(-29)
		Tonnersjoheden	May 12	May 3	(-9)	Apr 25	(-18)	Apr 4	(-39)
	Budburst 50%	Svartberget	Jun 17	Jun 8	(-9)	Jun 1	(-17)	May 23	(-26)
		Siljansfors	Jun 12	Jun 4	(-8)	May 27	(-16)	May 17	(-26)
		Asa	Jun 8	May 31	(-8)	May 25	(-15)	May 13	(-26)
		Tonnersjoheden	May 29	May 20	(-9)	May 14	(-15)	May 1	(-29)
	Budburst 90%	Svartberget	Jun 28	Jun 18	(-11)	Jun 11	(-18)	Jun 2	(-27)
		Siljansfors	Jun 26	Jun 17	(-10)	Jun 9	(-18)	May 30	(-28)
		Asa	Jun 30	Jun 21	(-9)	Jun 14	(-16)	Jun 3	(-27)
		Tonnersjoheden	Jun 15	Jun 6	(-9)	May 30	(-16)	May 18	(-28)
Norway spruce	Budburst 10%	Svartberget	May 29	May 21	(-8)	May 14	(-15)	May 5	(-24)
		Siljansfors	May 29	May 23	(-7)	May 16	(-13)	May 5	(-24)
		Asa	May 15	May 7	(-8)	May 1	(-15)	Apr 13	(-32)
		Tonnersjoheden	May 19	May 10	(-9)	May 3	(-16)	Apr 17	(-33)
	Budburst 50%	Svartberget	Jun 17	Jun 8	(-9)	Jun 1	(-17)	May 23	(-26)
		Siljansfors	Jun 15	Jun 7	(-8)	May 30	(-16)	May 19	(-27)
		Asa	Jun 10	Jun 2	(-8)	May 27	(-15)	May 15	(-26)
		Tonnersjoheden	Jun 7	May 29	(-9)	May 23	(-16)	May 10	(-28)
	Budburst 90%	Svartberget	Jul 5	Jun 25	(-11)	Jun 17	(-19)	Jun 8	(-27)
		Siljansfors	Jul 6	Jun 26	(-11)	Jun 17	(-20)	Jun 7	(-30)
		Asa	Jul 4	Jun 25	(-10)	Jun 18	(-17)	Jun 7	(-27)
		Tonnersjoheden	Jul 6	Jun 26	(-11)	Jun 19	(-18)	Jun 7	(-29)
Bilberry	Flowering > 10%	Svartberget	May 30	May 22	(-8)	May 15	(-15)	May 6	(-24)
		Siljansfors	May 27	May 21	(-7)	May 14	(-13)	May 3	(-24)
		Asa	May 14	May 6	(-8)	Apr 29	(-15)	Apr 10	(-34)
		Tonnersjoheden	May 7	Apr 28	(-10)	Apr 18	(-20)	Mar 23	(-46)
	Flowering Max	Svartberget	Jun 10	Jun 2	(-9)	May 26	(-16)	May 17	(-25)
		Siljansfors	May 29	May 23	(-7)	May 16	(-13)	May 5	(-24)
		Asa	May 23	May 16	(-8)	May 10	(-14)	Apr 26	(-28)
		Tonnersjoheden	May 18	May 10	(-9)	May 3	(-16)	Apr 16	(-33)
	Flowering<10%	Svartberget	Jun 25	Jun 16	(-10)	Jun 8	(-17)	May 30	(-26)
		Siljansfors	Jun 13	Jun 5	(-8)	May 28	(-16)	May 18	(-26)
		Asa	Jun 11	Jun 3	(-8)	May 28	(-15)	May 16	(-26)
		Tonnersjoheden	Jun 8	May 31	(-9)	May 24	(-16)	May 12	(-28)
	Ripe > 10%	Svartberget	Jul 28	Jul 14	(-14)	Jul 7	(-21)	Jun 27	(-32)
		Siljansfors	Jul 18	Jul 6	(-12)	Jun 28	(-21)	Jun 17	(-32)
		Asa	Jul 13	Jul 3	(-10)	Jun 27	(-17)	Jun 16	(-28)
		Tonnersjoheden	Jul 3	Jun 23	(-10)	Jun 16	(-18)	Jun 4	(-29)
	Ripe Max	Svartberget	Aug 15	Jul 28	(-18)	Jul 20	(-26)	Jul 8	(-38)
		Siljansfors	Aug 11	Jul 26	(-16)	Jul 17	(-26)	Jul 5	(-38)
		Asa	Aug 2	Jul 21	(-13)	Jul 14	(-20)	Jul 2	(-31)
		Tonnersjoheden	Jul 17	Jul 6	(-12)	Jun 29	(-19)	Jun 17	(-30)
Cowberry	Flowering > 10%	Svartberget	Jun 25	Jun 15	(-10)	Jun 8	(-17)	May 30	(-26)
		Siljansfors	Jun 9	Jun 2	(-8)	May 25	(-15)	May 15	(-26)
		Asa	May 28	May 20	(-8)	May 14	(-14)	May 2	(-27)
		Tonnersjoheden	May 26	May 18	(-9)	May 11	(-15)	Apr 27	(-29)
	Flowering Max	Svartberget	Jul 4	Jun 23	(-11)	Jun 16	(-18)	Jun 7	(-27)
		Siljansfors	Jun 22	Jun 13	(-9)	Jun 5	(-18)	May 26	(-28)
		Asa	Jun 21	Jun 12	(-9)	Jun 5	(-16)	May 25	(-27)
		Tonnersjoheden	Jun 11	Jun 2	(-9)	May 26	(-16)	May 14	(-28)
	Flowering < 10%	Svartberget	Jul 18	Jul 6	(-13)	Jun 29	(-20)	Jun 19	(-29)
		Siljansfors	Jul 8	Jun 28	(-11)	Jun 19	(-20)	Jun 9	(-30)
		Asa	Jul 15	Jul 5	(-11)	Jun 28	(-17)	Jun 17	(-28)
		Tonnersjoheden	Jul 2	Jun 22	(-10)	Jun 14	(-18)	Jun 3	(-29)
	Ripe > 10%	Svartberget	Aug 24	Aug 5	(-20)	Jul 27	(-29)	Jul 14	(-41)
		Siljansfors	Aug 17	Jul 31	(-18)	Jul 21	(-27)	Jul 8	(-40)
		Asa	Sep 3	Aug 14	(-21)	Aug 5	(-30)	Jul 22	(-44)
		Tonnersjoheden	Aug 7	Jul 25	(-14)	Jul 17	(-21)	Jul 5	(-33)
Spruce bark beetle	Generation 0	Svartberget	Jun 5	May 28	(-8)	May 22	(-15)	May 14	(-23)
		Siljansfors	May 25	May 20	(-6)	May 14	(-12)	May 4	(-22)
		Asa	May 20	May 14	(-6)	May 10	(-11)	May 1	(-20)
		Tonnersjoheden	May 14	May 8	(-6)	May 5	(-10)	Apr 25	(-19)
	Generation 1	Svartberget	Aug 16	Jul 30	(-18)	Jul 22	(-26)	Jul 9	(-39)
		Siljansfors	Aug 10	Jul 23	(-19)	Jul 13	(-29)	Jul 1	(-41)
		Asa	Aug 19	Jul 31	(-19)	Jul 24	(-27)	Jul 12	(-38)
		Tonnersjoheden	Aug 2	Jul 20	(-14)	Jul 13	(-21)	Jul 3	(-31)

**Fig. 8:**
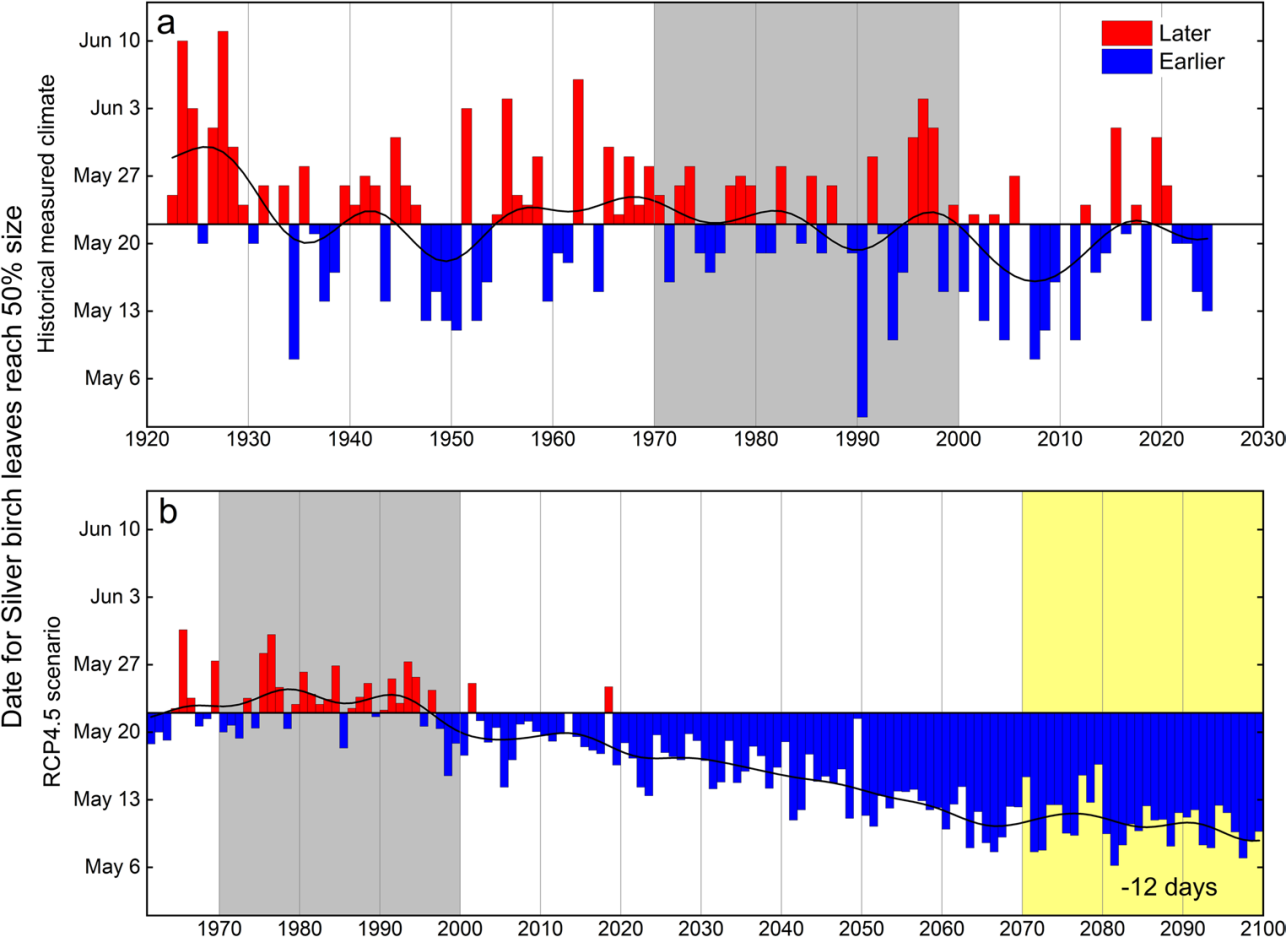
Estimated dates for leaves of Silver birch reaching 50% of its length, when the mean critical accumulated temperature for reaching this length at the site Siljansfors was applied (**a**) on historical daily average temperatures 1922–2024, and (**b**) on mean RCP4.5 scenario daily average temperatures (*n* = 17). Baseline was set to the mean date for the reference period 1970–1999 (grey area), which in both cases was set to May 22 and black lines show the moving average (5 years) of the dates. Target time frame is indicated with yellow, indicating the difference in days between the reference and target time frame mean dates

When comparing historical dates for phenological traits to occur, e.g. the date for leaves of Silver birch to reach 50% of their final size, using measured temperatures, to the scenarios, it was evident that no trends could be found back in time, but a clear trend was found in the future (Fig. 8). Notable is that the period in common of the two temperature datasets, the dates show a similar pattern of advanced and delayed dates, compared to the average date for the period 1970–1999, which was May 22 in both cases.

**Fig. 9:**
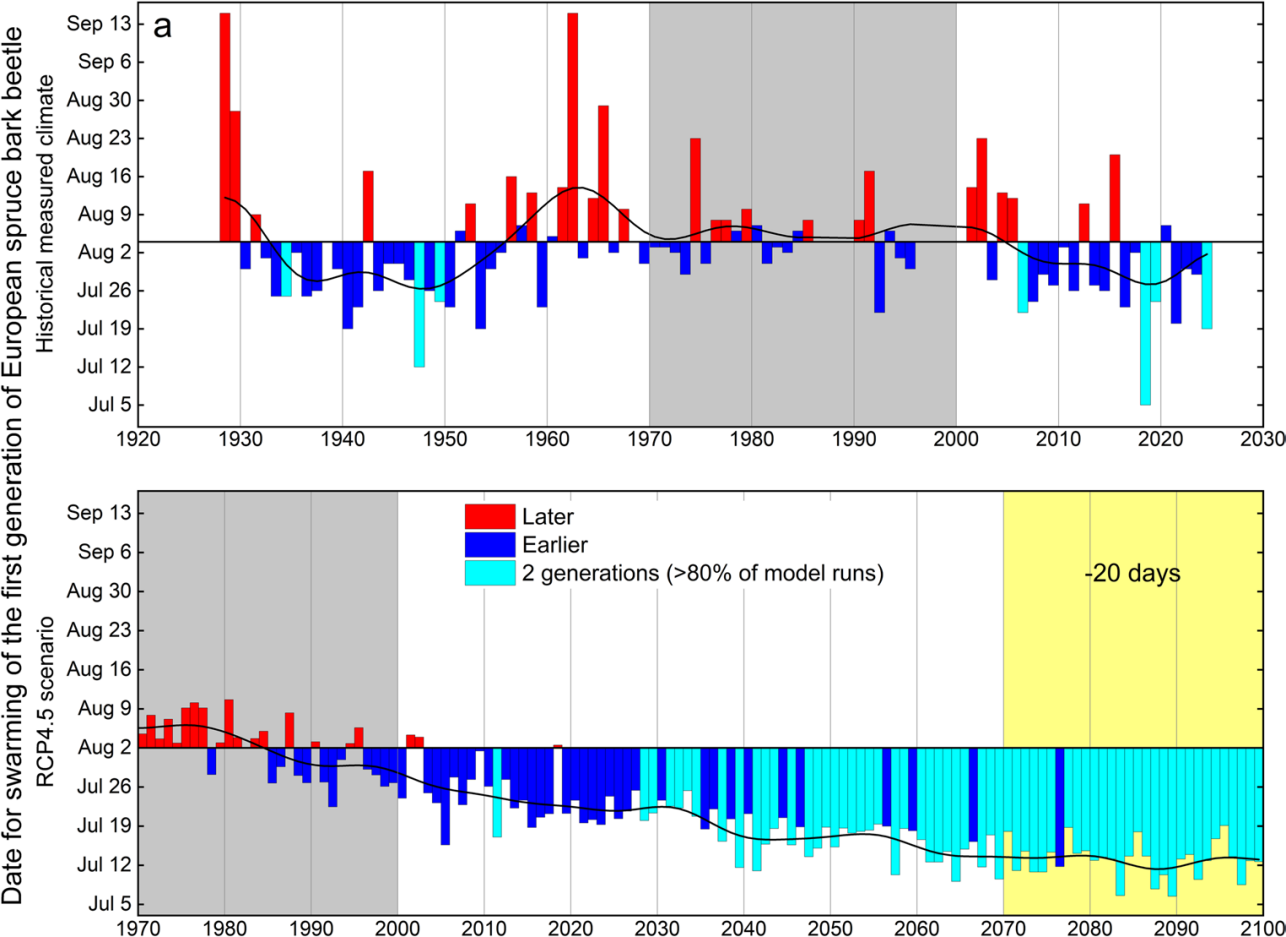
Estimated swarming dates for first generation European spruce bark beetle, i.e. when the mean critical accumulated temperature for swarming of the first generation of the year was reached at the site Tönnersjöheden (57°N), as applied on historical daily average temperatures (1928–2024), above, and on mean scenario (RCP4.5) daily average temperatures (*n* = 17), below. The baselines were based on the mean date for the years 1970–1999 (grey area), which was set to August 3 and August 2 for the historical and scenario data, respectively. Target time frame is indicated with yellow, indicating the difference in days between the reference and target time frame mean swarming dates. Years when temperatures allowed for a second generation to develop are indicated with turquoise bars (80% of the model runs)

When modelling the swarming of the European spruce bark beetle, the main result is that this phenology trait also shows a great advance in the swarming date of the first new generation of the year, in the future, 20 days on average for the period 2070–2099, at the site Tönnersjöheden (Fig. 9). This site has the highest temperatures in this study, depending on the low latitude and close distance to the west coast of Sweden, so the modelling for this site is the most extreme, when it comes to swarming conditions. This can be seen also when applied on the historical temperatures, showing that conditions for the bark beetle to be able to complete two new generations in one season has been fulfilled even during a few years back in time, with the most notable and also confirmed year in 2018. As seen in Fig. 9, the conditions for two generations in one season will be fulfilled more frequently in the future temperature climates, at least according to the stronger scenarios RCP4.5 and RCP8.5, and there were even found some model runs where three generations could be achieved. For the most pronounced year, 2068, risk for this to happen was rather high, as 41% of the model runs reached this level. The more continental and on higher latitudes, the risk for more than one new generation per season was lower, with the lowest risk found for the scenario RCP2.6 at the site Svartberget (64°).

## Discussion

### Forecast models

The short-term forecasts have been found to be important to predict the behavior of the traits studied during the current season. For example, the forecast of the flushing time of broadleaves is used for planning operations of airborne photography and laser scanning, as their outcome is highly dependent on whether the trees are leafed out or not. Similarly, the berry forecasts have been used by researchers, planning for studies of pollinating insects in the forest, as well as by berry pickers, when planning the optimal time for picking. Finally, the European spruce bark beetle forecasts are used by the Swedish Forest Agency, several forest companies and private forest owners, when planning sanitation and salvage logging of attacked trees, to avoid massive outbreaks in the future.

The tree forecast models predict the timing of a relative value of 10% advanced size of the bud/leaf. As an example, this stage was reached on April 16, 2025 for Silver birch in Siljansfors, compared to the estimate of April 13 (cf. Figure 5). This is not the same as budburst, as this was not seen in the visual assessments until April 29 on the same trees, but it is an indicator for when buds are initiating the growing season. The stage 50% developed size is clearly after the budburst, when the leaf is still expanding towards full size. This stage has a higher predictive performance, as estimates are based on interpolations between assessment readings with a large progress during that time and therefore is possible to get more distinct estimates that are comparable between years. The 90% stage is used instead of 100%, as the mean values of the yearly estimates tend to be diverging towards 100% rather than produce distinct values, when compared to the accumulated temperatures.

The use of post-diapause accumulated temperatures for the prediction of bark beetle swarming is well established (e.g. Hoffmann et al. 2025a, 2025b). The model used in this study has its weakest point in the estimate of the timing of the swarming by the fact that it also depends on the actual weather the days they swarm, which is generally during days where maximum air temperature reach + 18 °C and dry conditions prevails. This means that, even if the required temperature sum is reached earlier, they tend to swarm first when the current weather is feasible for swarming.

Note that all models show a general development of the phenological traits of the species in focus for each location. In reality, there is also a genetic variation within the species (e.g. Langvall et al. 2001) and a variation in microclimate for each assessment site within a location, depending on e.g. topography, soil moisture and tree layer conditions such as height, density, species (i.e., Langvall et al. 2001, 2002), so individuals of the same species may have quite advanced or delayed response to the reference climate measured at the locations.

### Scenario modeling

The average of all 17 model runs for all scenarios show an advance in phenology dates of all species and traits, so even the most modest scenario RCP2.6 predicts an effect of climate change that is substantial by the end of the century.

The advancement of bud, leaf and shoot development in the future could have a positive effect, as this could lead to a longer growing season for the forest and, thus, a potential increase in sequestration of carbon (Bergh et al. 2005, 2007). The advancement can also face a potential risk, as an earlier start of budburst may increase the risk for frost damage, as the number and severity of spring/early summer frost events may not decrease synchronously with the increased temperatures and (Lamichhane 2021; Liu et al. 2018), thus, not with the advance of budburst (Langvall 2011). Higher temperatures may also lead to an increased risk of drought events, as the evaporation increases, which can have the reverse effect of tree growth and, thus, lead to a decreased carbon sequestration (de Castro Segtowich 2025).

The earlier flowering of the forest berries also imposes a risk for increased frost damage of flowers, which in turn may lead to decreased yields of berries in the future. The modelled earlier ripening of berries could hardly be a risk *per se*, but warmer temperatures after the ripening occurs may also lead to earlier occurrence of overripe berries and, thus, a shorter picking period. It will then be even more important to keep track of the timing of the ripening and when the maximum number of ripe berries appears, e.g. with the berry forecast model presented here.

The scenarios concerning the European spruce bark beetle are predicting a challenging future for the Swedish spruce forest. The increased frequency of the appearance of two generations of beetles per season found here is already known from more southern latitudes of Europe (Hofmann et al. 2025a, b; Romashkin et al. 2020; Schebeck et al. 2022, 2024), but not from higher altitudes (Milosavljević et al. 2025). This may lead to more frequent and more intense outbreaks in Sweden, and has also been simulated for the future climate in the UK (Webb et al. 2025). A more successful rejuvenation of the beetle population in the north of Sweden would also lead to more severe damages to the spruce forest at higher latitudes and altitudes than we are used to, in the future (Jian et al. 2025).

The models used in this study is limited to only use air temperature as a forcing parameter, the accumulated temperature, as the driving variable. In general, this is the most important factor and has been found to be sufficient for the species and traits, and at the latitudes (57–64°N) studied here, at least during periods with the range of air temperatures found in the historical records for these sites (Langvall et al. 2019). This was illustrated when applying the models on the historical temperature data (cf. Figures 8 and 9). Even so, we can not be sure the predictions have the same accuracy and precision when using the models on scenario data, as they might have an extended range of temperatures that requires more complex models. Other potential driving factors could be dormancy break, using temperature for modelling chilling requirements (e.g. Fraga et al. 2021), and humidity, e.g. precipitation, air humidity, soil moisture (SMHI 2026). Their relations as driving factors for phenological traits seems to be somewhat more complex and, thus, more difficult to implement in the scenario models (e.g. Nanninga et al. 2026; Shaozhi et al. 2022). These results should only be interpreted as indicators of the trends we can foresee for the future, but as these trends are rather strong, especially for the two scenarios RCP4.5 and RCP8.5, they are still strong indicators of the climate change effects on the Swedish nature.

## Conclusions

Forecast models for forest phenology are shown to be useful tools for the seasonal planning of activities within the areal industries and the public. They are especially valuable on years deviating much from the normal year, as they then can be used to adjust the timing of activities in the forest that are dependent on the timing of certain phenological traits, e.g. when staff for picking berries should be hired.

It is hard to specify more exact findings for scenario analyses, as scenarios are just speculations of the future climate and this is our “best guess” of what would happen, if climate change develop as the scenarios describe. Generally, we did not find any trends when applying the models on historical weather data, but a strong advancing trend in the timing of all studied forest phenology responses to a future climate, particularly if climate change takes the pathway similar to the scenario RCP8.5. This was especially clear, when modelling the European spruce bark beetle, as the frequency of years when the conditions for producing more than one new generation of beetles increased so that, at the end of the century, we can expect two generations per season to be the new normal condition.

## Data Availability

Forecast model data for tree and berry phenology are available at the repository www.researchdata.se with the identifier 10.5878/jbab-cy46. The basis for the forecast model of European bark beetle swarming phenology are available on request from the authors Fritscher and Schroeder (2022). Climate data from the Experimental Forests used in the forecast models are available at the following URL: https://esf-klimatdata.slu.se/QC_klimatdata.aspx?lang=en. The general trends of climate scenarios for Sweden can be seen at the URL: https://www.smhi.se/klimat/framtidens-klimat/klimatscenariotjansten/klimatscenariotjansten/hyd/-/effektivnederbord/rcp45/2071-2100/jul/unregulated. Single model run climate scenario data for certain grid points in Sweden, as used in this paper, are available on request from the Swedish Meteorological and Hydrological Institute (www.smhi.se).
